# A triclinic polymorph of (*E*)-2-(2-nitro­ethen­yl)furan

**DOI:** 10.1107/S160053681002492X

**Published:** 2010-07-03

**Authors:** Lin Li, Bo Yu

**Affiliations:** a Key Laboratory of Catalysis and Materials Science of the State Ethnic Affairs Commission & Ministry of Education, College of Chemistry and Materials Science, South-Central University for Nationalities, Wuhan 430074, People’s Republic of China

## Abstract

The title compound, C_6_H_5_NO_3_, crystallizes in the triclinic system with six independent mol­ecules in the asymmetric unit. In a previous study, the structure of the title compound was determined in the monoclinic *P*2_1_/*n* space group at 100 K [Valerga *et al.* (2009 ▶). *Acta Cryst.* E**65**, o1979]. All six independent mol­ecules display an *E* configuration about the C=C double bond, with the dihedral angles between the planes of the furan rings and the nitro­alkenyl groups ranging from 0.61 (7) to 5.03 (7)°. The crystal structure is stabilized by inter­molecular C—H⋯O hydrogen-bonding inter­actions.

## Related literature

For the use of nitro­alkenes in organic synthesis, see: Ranu *et al.* (2005 ▶); Ballini & Bosica (2005 ▶); Ono (2005 ▶). For the structure of the monoclinic polymorph, see: Valerga *et al.* (2009 ▶).
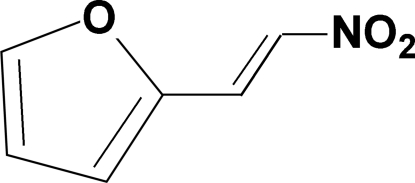


## Experimental

### 

#### Crystal data

C_6_H_5_NO_3_
*M* *_r_* = 139.11
Triclinic, 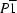

*a* = 9.8407 (14) Å
*b* = 13.4270 (19) Å
*c* = 15.300 (2) Å
α = 91.105 (1)°
β = 108.603 (2)°
γ = 91.172 (1)°
*V* = 1914.8 (5) Å^3^
*Z* = 12
Mo *K*α radiation
μ = 0.12 mm^−1^
*T* = 298 K
0.12 × 0.10 × 0.10 mm

#### Data collection

Bruker SMART CCD area-detector diffractometer
Absorption correction: multi-scan (*SADABS*; Sheldrick, 1997 ▶) *T*_min_ = 0.976, *T*_max_ = 0.988
13307 measured reflections
8182 independent reflections
4235 reflections with *I* > 2σ(*I*)
*R*_int_ = 0.027

#### Refinement

*R*[*F* ^2^ > 2σ(*F* ^2^)] = 0.058
*wR*(*F* ^2^) = 0.149
*S* = 0.92
8182 reflections
541 parameters
H-atom parameters constrained
Δρ_max_ = 0.15 e Å^−3^
Δρ_min_ = −0.22 e Å^−3^

### 

Data collection: *SMART* (Bruker, 2001 ▶); cell refinement: *SAINT-Plus* (Bruker, 2001 ▶); data reduction: *SAINT-Plus*; program(s) used to solve structure: *SHELXS97* (Sheldrick, 2008 ▶); program(s) used to refine structure: *SHELXL97* (Sheldrick, 2008 ▶); molecular graphics: *PLATON* (Spek, 2009 ▶); software used to prepare material for publication: *PLATON*.

## Supplementary Material

Crystal structure: contains datablocks global, I. DOI: 10.1107/S160053681002492X/rz2466sup1.cif

Structure factors: contains datablocks I. DOI: 10.1107/S160053681002492X/rz2466Isup2.hkl

Additional supplementary materials:  crystallographic information; 3D view; checkCIF report

## Figures and Tables

**Table 1:** Hydrogen-bond geometry (Å, °)

*D*—H⋯*A*	*D*—H	H⋯*A*	*D*⋯*A*	*D*—H⋯*A*
C1—H1⋯O10^i^	0.93	2.58	3.382 (3)	145
C16—H16⋯O13	0.93	2.60	3.370 (3)	141
C26—H26⋯O14^ii^	0.93	2.59	3.281 (3)	131
C28—H28⋯O8^iii^	0.93	2.59	3.405 (3)	146
C34—H34⋯O4	0.93	2.59	3.383 (3)	143

Symmetry codes: (i) 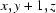; (ii) 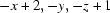; (iii) 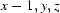.

**Fig. 1.**
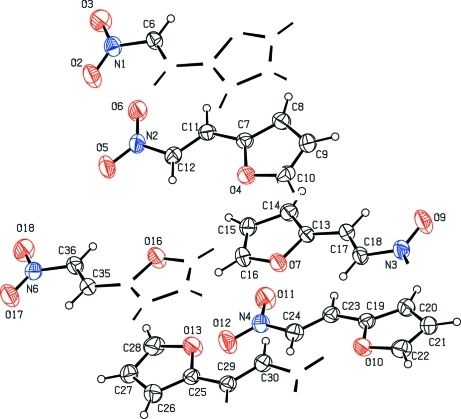
The asymmetric unit of the title compound showing the atom-numbering scheme. Displacement ellipsoids are drawn at the 30% probability level.

**Fig. 2.**
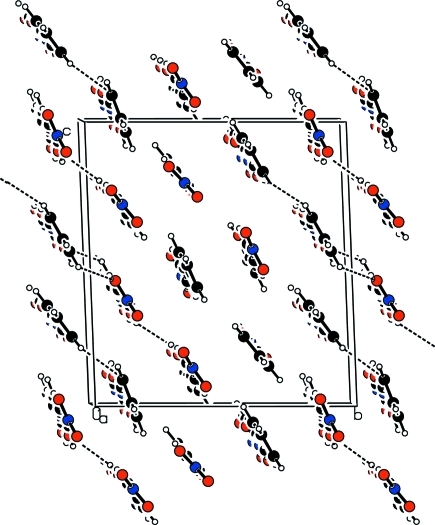
Crystal packing for the title compound, with C—H···O interactions drawn as dashed lines.

## supplementary crystallographic information

### Comment

Nitroalkenes are good substrates for Michael addition reactions because of the stronger electrowithdrawing property of the nitro group (Ranu *et al.*, 2005). At the same time, the nitro group can provide a good nitrogen source for the synthesis of many useful organic molecules (Ballini & Bosica, 2005; Ono, 2005). Our group focus on new organic transformations obtained by nitroalkene as substrates. In this paper, we report the structure of a triclinic polymorph of the title compound. Recently, the structure of the title compound was determined in the monoclinic *P*2_1_/*n* space group at 100 K (Valerga *et al.*, 2009).

In the asymmetric unit of the title compound, there are six independent molecules (Fig.1). All molecules display an *E* configuration about the C═C double bond. Bond lengths and angles are in normal ranges and are comparable with those found in the monoclinic polymorph. The dihedral angles between the planes of the furan rings and the nitroalkenyl groups range from 0.61 (7) to 5.03 (7)°. The crystal structure (Fig. 2) is stabilized by intermolecular C—H···O hydrogen bonding interactions (Table 1).

### Experimental

Furfural (0.1 mol) and nitromethane (0.1 mol) were dissolved in 30 ml CH_3_OH with stirring under ice bath, and a few ml of a NaOH in CH_3_OH solution was added dropwise. After stirring for 1 h, ice water was added and the solution neutralized with a diluted hydrochloric acid solution. The yellowish-brown solid precipitate was filtered and recrystallized from C_2_H_5_OH (yield  92%). Crystals suitable for X-ray analysis were obtained by slow evaporation of a dichloromethane/hexane (1:1 *v*/*v*) solution at 283 K.

### Refinement

All hydrogen atoms were placed in geometrically idealized positions with C–H = 0.93 Å, and constrained to ride on their parent atoms with *U*_iso_(H) = 1.2 *U*_eq_(C).

### Crystal data

**Table:**

C_6_H_5_NO_3_	*Z* = 12
*M**_r_* = 139.11	*F*(000) = 864
Triclinic, *P*1	*D*_x_ = 1.448 Mg m^−^^3^
Hall symbol: -P 1	Mo *K*α radiation, λ = 0.71073 Å
*a* = 9.8407 (14) Å	Cell parameters from 2345 reflections
*b* = 13.4270 (19) Å	θ = 1.2–25.9°
*c* = 15.300 (2) Å	µ = 0.12 mm^−^^1^
α = 91.105 (1)°	*T* = 298 K
β = 108.603 (2)°	Block, colourless
γ = 91.172 (1)°	0.12 × 0.10 × 0.10 mm
*V* = 1914.8 (5) Å^3^	

### Data collection

**Table:**

Bruker SMART CCD area-detector diffractometer	8182 independent reflections
Radiation source: fine-focus sealed tube	4235 reflections with *I* > 2σ(*I*)
graphite	*R*_int_ = 0.027
φ and ω scans	θ_max_ = 27.0°, θ_min_ = 2.0°
Absorption correction: multi-scan (*SADABS*; Sheldrick, 1997)	*h* = −12→12
*T*_min_ = 0.976, *T*_max_ = 0.988	*k* = −16→17
13307 measured reflections	*l* = −14→19

### Refinement

**Table:**

Refinement on *F*^2^	Primary atom site location: structure-invariant direct methods
Least-squares matrix: full	Secondary atom site location: difference Fourier map
*R*[*F*^2^ > 2σ(*F*^2^)] = 0.058	Hydrogen site location: inferred from neighbouring sites
*wR*(*F*^2^) = 0.149	H-atom parameters constrained
*S* = 0.92	*w* = 1/[σ^2^(*F*_o_^2^) + (0.0705*P*)^2^] where *P* = (*F*_o_^2^ + 2*F*_c_^2^)/3
8182 reflections	(Δ/σ)_max_ = 0.002
541 parameters	Δρ_max_ = 0.15 e Å^−^^3^
0 restraints	Δρ_min_ = −0.22 e Å^−^^3^

### Special details

**Table:**

Geometry. All e.s.d.'s (except the e.s.d. in the dihedral angle between two l.s. planes) are estimated using the full covariance matrix. The cell e.s.d.'s are taken into account individually in the estimation of e.s.d.'s in distances, angles and torsion angles; correlations between e.s.d.'s in cell parameters are only used when they are defined by crystal symmetry. An approximate (isotropic) treatment of cell e.s.d.'s is used for estimating e.s.d.'s involving l.s. planes.
Refinement. Refinement of *F*^2^ against ALL reflections. The weighted *R*-factor *wR* and goodness of fit *S* are based on *F*^2^, conventional *R*-factors *R* are based on *F*, with *F* set to zero for negative *F*^2^. The threshold expression of *F*^2^ > σ(*F*^2^) is used only for calculating *R*-factors(gt) *etc*. and is not relevant to the choice of reflections for refinement. *R*-factors based on *F*^2^ are statistically about twice as large as those based on *F*, and *R*- factors based on ALL data will be even larger.

### Fractional atomic coordinates and isotropic or equivalent isotropic displacement parameters (Å^2^)

**Table:**

	*x*	*y*	*z*	*U*_iso_*/*U*_eq_	
C1	0.9416 (2)	0.92135 (16)	0.24460 (16)	0.0650 (6)	
H1	0.9831	0.9532	0.2056	0.078*	
C2	1.0123 (2)	0.86624 (16)	0.31429 (16)	0.0644 (6)	
H2	1.1096	0.8528	0.3329	0.077*	
C3	0.9112 (2)	0.83226 (15)	0.35424 (15)	0.0599 (6)	
H3	0.9288	0.7912	0.4047	0.072*	
C4	0.7841 (2)	0.86944 (14)	0.30671 (14)	0.0479 (5)	
C5	0.6460 (2)	0.85930 (14)	0.31761 (14)	0.0517 (5)	
H5	0.6387	0.8211	0.3661	0.062*	
C6	0.5279 (2)	0.89934 (15)	0.26493 (14)	0.0539 (5)	
H6	0.5308	0.9382	0.2159	0.065*	
C7	0.7237 (2)	0.63452 (14)	0.18843 (14)	0.0491 (5)	
C8	0.8029 (2)	0.67212 (15)	0.13981 (16)	0.0638 (6)	
H8	0.7714	0.7146	0.0905	0.077*	
C9	0.9416 (2)	0.63636 (16)	0.17613 (17)	0.0669 (7)	
H9	1.0196	0.6501	0.1560	0.080*	
C10	0.9392 (2)	0.57880 (16)	0.24518 (17)	0.0657 (6)	
H10	1.0178	0.5452	0.2819	0.079*	
C11	0.5770 (2)	0.64540 (14)	0.18134 (14)	0.0515 (5)	
H11	0.5239	0.6880	0.1368	0.062*	
C12	0.5094 (2)	0.60051 (15)	0.23223 (15)	0.0567 (6)	
H12	0.5587	0.5573	0.2776	0.068*	
C13	0.8059 (2)	0.37791 (14)	0.10512 (14)	0.0484 (5)	
C14	0.6775 (2)	0.40843 (14)	0.05235 (15)	0.0570 (6)	
H14	0.6596	0.4424	−0.0024	0.068*	
C15	0.5752 (2)	0.38043 (15)	0.09389 (16)	0.0599 (6)	
H15	0.4772	0.3915	0.0726	0.072*	
C16	0.6481 (2)	0.33457 (16)	0.17040 (16)	0.0643 (6)	
H16	0.6070	0.3081	0.2121	0.077*	
C17	0.9454 (2)	0.38754 (14)	0.09616 (14)	0.0498 (5)	
H17	0.9525	0.4201	0.0446	0.060*	
C18	1.0657 (2)	0.35470 (14)	0.15413 (14)	0.0525 (5)	
H18	1.0631	0.3202	0.2057	0.063*	
C19	0.8813 (2)	0.13701 (14)	0.01911 (15)	0.0495 (5)	
C20	0.9605 (2)	0.15719 (15)	−0.03563 (16)	0.0607 (6)	
H20	0.9267	0.1808	−0.0953	0.073*	
C21	1.1038 (2)	0.13626 (15)	0.01342 (18)	0.0660 (6)	
H21	1.1826	0.1428	−0.0073	0.079*	
C22	1.1038 (2)	0.10540 (16)	0.09513 (18)	0.0697 (7)	
H22	1.1852	0.0868	0.1418	0.084*	
C23	0.7319 (2)	0.14092 (14)	0.00502 (15)	0.0512 (5)	
H23	0.6750	0.1662	−0.0504	0.061*	
C24	0.6664 (2)	0.11183 (15)	0.06361 (15)	0.0565 (6)	
H24	0.7199	0.0866	0.1200	0.068*	
C25	0.7166 (2)	0.11542 (14)	0.39786 (15)	0.0505 (5)	
C26	0.6371 (2)	0.08594 (15)	0.45004 (16)	0.0640 (6)	
H26	0.6703	0.0533	0.5056	0.077*	
C27	0.4949 (2)	0.11306 (16)	0.40575 (18)	0.0675 (7)	
H27	0.4161	0.1022	0.4258	0.081*	
C28	0.4967 (2)	0.15721 (17)	0.32982 (19)	0.0766 (7)	
H28	0.4164	0.1829	0.2868	0.092*	
C29	0.8654 (2)	0.10866 (14)	0.40970 (14)	0.0512 (5)	
H29	0.9203	0.0741	0.4604	0.061*	
C30	0.9330 (2)	0.14664 (15)	0.35584 (15)	0.0564 (6)	
H30	0.8818	0.1819	0.3046	0.068*	
C31	0.6315 (2)	0.36980 (14)	0.48376 (15)	0.0505 (5)	
C32	0.7617 (2)	0.34777 (16)	0.53904 (15)	0.0635 (6)	
H32	0.7822	0.3193	0.5966	0.076*	
C33	0.8624 (2)	0.37469 (15)	0.49599 (17)	0.0628 (6)	
H33	0.9613	0.3678	0.5186	0.075*	
C34	0.7873 (3)	0.41190 (17)	0.41671 (17)	0.0739 (7)	
H34	0.8270	0.4361	0.3734	0.089*	
C35	0.4923 (2)	0.35871 (14)	0.49243 (14)	0.0522 (5)	
H35	0.4868	0.3295	0.5459	0.063*	
C36	0.3707 (2)	0.38543 (15)	0.43283 (15)	0.0565 (6)	
H36	0.3716	0.4150	0.3785	0.068*	
N1	0.39430 (19)	0.88338 (14)	0.28277 (13)	0.0603 (5)	
N2	0.3605 (2)	0.61778 (13)	0.21816 (13)	0.0608 (5)	
N3	1.19984 (19)	0.37178 (13)	0.13825 (14)	0.0597 (5)	
N4	0.51390 (19)	0.11858 (12)	0.04148 (14)	0.0589 (5)	
N5	1.08370 (18)	0.13472 (13)	0.37455 (13)	0.0574 (5)	
N6	0.23663 (19)	0.36992 (13)	0.44974 (14)	0.0600 (5)	
O1	0.80031 (15)	0.92554 (10)	0.23725 (10)	0.0586 (4)	
O2	0.38865 (17)	0.82959 (14)	0.34471 (12)	0.0853 (5)	
O3	0.29023 (16)	0.92505 (12)	0.23200 (12)	0.0870 (5)	
O4	0.80683 (15)	0.57538 (10)	0.25532 (10)	0.0614 (4)	
O5	0.30358 (18)	0.57248 (13)	0.26641 (12)	0.0847 (5)	
O6	0.29579 (16)	0.67661 (12)	0.16014 (11)	0.0804 (5)	
O7	0.79022 (15)	0.33117 (10)	0.17990 (9)	0.0588 (4)	
O8	1.30633 (16)	0.33720 (12)	0.19333 (12)	0.0844 (5)	
O9	1.20458 (17)	0.41996 (14)	0.07243 (12)	0.0877 (6)	
O10	0.96849 (15)	0.10452 (10)	0.10184 (10)	0.0629 (4)	
O11	0.44264 (16)	0.15312 (12)	−0.03166 (11)	0.0803 (5)	
O12	0.46063 (17)	0.08859 (13)	0.09852 (12)	0.0849 (5)	
O13	0.63096 (15)	0.16044 (11)	0.32253 (11)	0.0711 (5)	
O14	1.15176 (16)	0.09038 (12)	0.44346 (11)	0.0782 (5)	
O15	1.13827 (17)	0.17002 (12)	0.32070 (12)	0.0805 (5)	
O16	0.64420 (16)	0.41055 (11)	0.40583 (10)	0.0709 (5)	
O17	0.23464 (18)	0.33222 (13)	0.52140 (13)	0.0894 (6)	
O18	0.12831 (16)	0.39605 (12)	0.39036 (11)	0.0817 (5)	

### Atomic displacement parameters (Å^2^)

**Table:**

	*U*^11^	*U*^22^	*U*^33^	*U*^12^	*U*^13^	*U*^23^
C1	0.0618 (16)	0.0695 (15)	0.0780 (17)	−0.0058 (12)	0.0427 (14)	0.0025 (13)
C2	0.0469 (13)	0.0728 (15)	0.0772 (17)	0.0049 (11)	0.0246 (13)	0.0062 (13)
C3	0.0560 (14)	0.0666 (14)	0.0570 (14)	0.0048 (11)	0.0171 (12)	0.0129 (11)
C4	0.0490 (13)	0.0499 (12)	0.0475 (13)	−0.0027 (9)	0.0190 (10)	0.0047 (10)
C5	0.0531 (13)	0.0529 (12)	0.0511 (13)	−0.0015 (10)	0.0195 (11)	0.0040 (10)
C6	0.0502 (13)	0.0580 (13)	0.0565 (14)	−0.0032 (10)	0.0215 (11)	0.0050 (10)
C7	0.0463 (12)	0.0480 (12)	0.0522 (13)	0.0049 (9)	0.0142 (10)	0.0078 (10)
C8	0.0573 (14)	0.0591 (14)	0.0791 (17)	0.0037 (11)	0.0264 (13)	0.0173 (12)
C9	0.0541 (15)	0.0621 (15)	0.0925 (19)	0.0008 (11)	0.0344 (14)	0.0078 (13)
C10	0.0481 (14)	0.0652 (15)	0.0800 (18)	0.0130 (11)	0.0144 (12)	0.0033 (13)
C11	0.0502 (13)	0.0460 (12)	0.0575 (14)	0.0037 (9)	0.0158 (11)	0.0053 (10)
C12	0.0507 (13)	0.0602 (13)	0.0621 (15)	0.0135 (10)	0.0207 (12)	0.0092 (11)
C13	0.0480 (12)	0.0481 (12)	0.0525 (13)	−0.0016 (9)	0.0210 (10)	0.0071 (10)
C14	0.0487 (13)	0.0608 (13)	0.0608 (14)	0.0065 (10)	0.0156 (11)	0.0135 (11)
C15	0.0432 (12)	0.0628 (14)	0.0753 (16)	0.0035 (10)	0.0208 (12)	0.0050 (12)
C16	0.0562 (15)	0.0691 (15)	0.0791 (17)	−0.0022 (11)	0.0378 (13)	0.0097 (13)
C17	0.0507 (13)	0.0516 (12)	0.0514 (13)	−0.0012 (10)	0.0223 (10)	0.0042 (10)
C18	0.0462 (12)	0.0584 (13)	0.0573 (14)	−0.0003 (10)	0.0226 (11)	0.0071 (10)
C19	0.0449 (12)	0.0456 (12)	0.0581 (14)	0.0041 (9)	0.0161 (11)	0.0067 (10)
C20	0.0558 (14)	0.0641 (14)	0.0666 (15)	0.0086 (11)	0.0244 (12)	0.0153 (12)
C21	0.0523 (14)	0.0609 (14)	0.0933 (19)	0.0058 (11)	0.0343 (14)	0.0140 (13)
C22	0.0402 (13)	0.0669 (15)	0.097 (2)	0.0073 (10)	0.0131 (13)	0.0213 (14)
C23	0.0449 (12)	0.0487 (12)	0.0589 (14)	0.0041 (9)	0.0147 (11)	0.0060 (10)
C24	0.0408 (12)	0.0586 (13)	0.0652 (15)	0.0022 (10)	0.0100 (11)	0.0069 (11)
C25	0.0444 (12)	0.0465 (12)	0.0606 (14)	0.0040 (9)	0.0165 (11)	0.0053 (10)
C26	0.0591 (15)	0.0654 (15)	0.0714 (16)	0.0058 (11)	0.0258 (13)	0.0098 (12)
C27	0.0523 (14)	0.0587 (14)	0.099 (2)	0.0009 (11)	0.0341 (14)	0.0041 (13)
C28	0.0416 (13)	0.0735 (16)	0.110 (2)	0.0120 (11)	0.0151 (14)	0.0239 (15)
C29	0.0441 (12)	0.0502 (12)	0.0580 (14)	0.0041 (9)	0.0140 (10)	0.0055 (10)
C30	0.0418 (12)	0.0607 (13)	0.0644 (15)	0.0063 (10)	0.0130 (11)	0.0113 (11)
C31	0.0547 (14)	0.0463 (12)	0.0535 (14)	−0.0002 (10)	0.0212 (11)	0.0068 (10)
C32	0.0570 (14)	0.0734 (15)	0.0646 (15)	0.0101 (11)	0.0241 (13)	0.0195 (12)
C33	0.0493 (13)	0.0651 (14)	0.0773 (17)	0.0053 (11)	0.0241 (13)	0.0112 (12)
C34	0.0607 (16)	0.0906 (18)	0.0818 (19)	−0.0017 (13)	0.0385 (14)	0.0167 (14)
C35	0.0546 (13)	0.0515 (12)	0.0549 (14)	0.0029 (10)	0.0232 (11)	0.0059 (10)
C36	0.0501 (13)	0.0594 (13)	0.0638 (15)	−0.0009 (10)	0.0233 (12)	0.0082 (11)
N1	0.0445 (11)	0.0722 (13)	0.0635 (13)	−0.0022 (9)	0.0162 (10)	0.0023 (10)
N2	0.0531 (12)	0.0695 (13)	0.0664 (13)	0.0092 (10)	0.0277 (10)	0.0066 (10)
N3	0.0474 (11)	0.0686 (12)	0.0667 (13)	0.0013 (9)	0.0232 (10)	0.0070 (10)
N4	0.0449 (11)	0.0592 (12)	0.0732 (14)	0.0007 (9)	0.0194 (10)	0.0058 (10)
N5	0.0436 (11)	0.0605 (12)	0.0689 (14)	0.0040 (9)	0.0186 (10)	0.0046 (10)
N6	0.0503 (12)	0.0578 (12)	0.0761 (14)	0.0038 (9)	0.0257 (11)	0.0066 (10)
O1	0.0573 (9)	0.0610 (9)	0.0617 (10)	0.0035 (7)	0.0243 (8)	0.0130 (7)
O2	0.0609 (11)	0.1205 (14)	0.0839 (13)	0.0062 (10)	0.0338 (9)	0.0390 (11)
O3	0.0484 (10)	0.1061 (13)	0.1021 (13)	0.0130 (9)	0.0155 (9)	0.0318 (10)
O4	0.0534 (9)	0.0672 (10)	0.0656 (10)	0.0106 (7)	0.0203 (8)	0.0171 (8)
O5	0.0710 (11)	0.1055 (13)	0.0971 (13)	0.0118 (10)	0.0522 (10)	0.0297 (10)
O6	0.0586 (10)	0.1003 (13)	0.0887 (12)	0.0279 (9)	0.0290 (9)	0.0350 (10)
O7	0.0507 (9)	0.0679 (10)	0.0616 (10)	0.0032 (7)	0.0220 (7)	0.0174 (8)
O8	0.0466 (9)	0.1086 (13)	0.0965 (13)	0.0181 (9)	0.0180 (9)	0.0305 (10)
O9	0.0608 (11)	0.1255 (15)	0.0889 (13)	0.0044 (10)	0.0385 (10)	0.0417 (11)
O10	0.0447 (9)	0.0705 (10)	0.0728 (11)	0.0043 (7)	0.0161 (8)	0.0234 (8)
O11	0.0480 (9)	0.1092 (13)	0.0791 (12)	0.0135 (9)	0.0119 (9)	0.0261 (10)
O12	0.0587 (11)	0.1081 (14)	0.0985 (14)	−0.0004 (9)	0.0386 (10)	0.0290 (11)
O13	0.0428 (9)	0.0858 (11)	0.0836 (12)	0.0072 (8)	0.0167 (8)	0.0322 (9)
O14	0.0495 (9)	0.1025 (13)	0.0824 (12)	0.0191 (9)	0.0184 (9)	0.0287 (10)
O15	0.0602 (10)	0.1047 (13)	0.0902 (13)	0.0043 (9)	0.0414 (10)	0.0295 (10)
O16	0.0574 (10)	0.0928 (12)	0.0663 (11)	0.0032 (8)	0.0235 (8)	0.0250 (9)
O17	0.0698 (12)	0.1143 (14)	0.0977 (14)	0.0103 (10)	0.0431 (11)	0.0430 (11)
O18	0.0482 (10)	0.1026 (13)	0.0895 (13)	0.0134 (9)	0.0139 (9)	0.0155 (10)

### Geometric parameters (Å, °)

**Table:**

C1—C2	1.321 (3)	C21—H21	0.9300
C1—O1	1.362 (2)	C22—O10	1.368 (3)
C1—H1	0.9300	C22—H22	0.9300
C2—C3	1.397 (3)	C23—C24	1.320 (3)
C2—H2	0.9300	C23—H23	0.9300
C3—C4	1.342 (3)	C24—N4	1.434 (2)
C3—H3	0.9300	C24—H24	0.9300
C4—O1	1.362 (2)	C25—C26	1.343 (3)
C4—C5	1.425 (3)	C25—O13	1.353 (2)
C5—C6	1.317 (3)	C25—C29	1.422 (3)
C5—H5	0.9300	C26—C27	1.405 (3)
C6—N1	1.438 (3)	C26—H26	0.9300
C6—H6	0.9300	C27—C28	1.319 (3)
C7—C8	1.336 (3)	C27—H27	0.9300
C7—O4	1.368 (2)	C28—O13	1.361 (3)
C7—C11	1.424 (3)	C28—H28	0.9300
C8—C9	1.398 (3)	C29—C30	1.317 (3)
C8—H8	0.9300	C29—H29	0.9300
C9—C10	1.327 (3)	C30—N5	1.431 (2)
C9—H9	0.9300	C30—H30	0.9300
C10—O4	1.360 (2)	C31—C32	1.334 (3)
C10—H10	0.9300	C31—O16	1.362 (2)
C11—C12	1.320 (3)	C31—C35	1.423 (3)
C11—H11	0.9300	C32—C33	1.399 (3)
C12—N2	1.436 (3)	C32—H32	0.9300
C12—H12	0.9300	C33—C34	1.317 (3)
C13—C14	1.341 (3)	C33—H33	0.9300
C13—O7	1.365 (2)	C34—O16	1.364 (3)
C13—C17	1.426 (3)	C34—H34	0.9300
C14—C15	1.401 (3)	C35—C36	1.314 (3)
C14—H14	0.9300	C35—H35	0.9300
C15—C16	1.332 (3)	C36—N6	1.435 (3)
C15—H15	0.9300	C36—H36	0.9300
C16—O7	1.361 (2)	N1—O2	1.217 (2)
C16—H16	0.9300	N1—O3	1.224 (2)
C17—C18	1.323 (3)	N2—O5	1.223 (2)
C17—H17	0.9300	N2—O6	1.226 (2)
C18—N3	1.431 (3)	N3—O9	1.220 (2)
C18—H18	0.9300	N3—O8	1.223 (2)
C19—C20	1.341 (3)	N4—O11	1.221 (2)
C19—O10	1.368 (2)	N4—O12	1.223 (2)
C19—C23	1.419 (3)	N5—O15	1.216 (2)
C20—C21	1.407 (3)	N5—O14	1.227 (2)
C20—H20	0.9300	N6—O17	1.222 (2)
C21—C22	1.325 (3)	N6—O18	1.222 (2)
			
C2—C1—O1	111.4 (2)	C24—C23—C19	125.8 (2)
C2—C1—H1	124.3	C24—C23—H23	117.1
O1—C1—H1	124.3	C19—C23—H23	117.1
C1—C2—C3	106.0 (2)	C23—C24—N4	120.9 (2)
C1—C2—H2	127.0	C23—C24—H24	119.5
C3—C2—H2	127.0	N4—C24—H24	119.5
C4—C3—C2	107.74 (19)	C26—C25—O13	108.90 (18)
C4—C3—H3	126.1	C26—C25—C29	132.1 (2)
C2—C3—H3	126.1	O13—C25—C29	119.0 (2)
C3—C4—O1	109.20 (18)	C25—C26—C27	107.9 (2)
C3—C4—C5	131.5 (2)	C25—C26—H26	126.0
O1—C4—C5	119.26 (19)	C27—C26—H26	126.0
C6—C5—C4	125.6 (2)	C28—C27—C26	105.5 (2)
C6—C5—H5	117.2	C28—C27—H27	127.2
C4—C5—H5	117.2	C26—C27—H27	127.2
C5—C6—N1	120.4 (2)	C27—C28—O13	111.4 (2)
C5—C6—H6	119.8	C27—C28—H28	124.3
N1—C6—H6	119.8	O13—C28—H28	124.3
C8—C7—O4	109.22 (18)	C30—C29—C25	126.3 (2)
C8—C7—C11	131.9 (2)	C30—C29—H29	116.9
O4—C7—C11	118.83 (19)	C25—C29—H29	116.9
C7—C8—C9	107.9 (2)	C29—C30—N5	121.3 (2)
C7—C8—H8	126.0	C29—C30—H30	119.3
C9—C8—H8	126.0	N5—C30—H30	119.3
C10—C9—C8	106.0 (2)	C32—C31—O16	108.79 (19)
C10—C9—H9	127.0	C32—C31—C35	132.6 (2)
C8—C9—H9	127.0	O16—C31—C35	118.58 (19)
C9—C10—O4	111.2 (2)	C31—C32—C33	108.6 (2)
C9—C10—H10	124.4	C31—C32—H32	125.7
O4—C10—H10	124.4	C33—C32—H32	125.7
C12—C11—C7	126.0 (2)	C34—C33—C32	105.3 (2)
C12—C11—H11	117.0	C34—C33—H33	127.4
C7—C11—H11	117.0	C32—C33—H33	127.4
C11—C12—N2	120.57 (19)	C33—C34—O16	111.7 (2)
C11—C12—H12	119.7	C33—C34—H34	124.1
N2—C12—H12	119.7	O16—C34—H34	124.1
C14—C13—O7	109.09 (18)	C36—C35—C31	126.8 (2)
C14—C13—C17	131.9 (2)	C36—C35—H35	116.6
O7—C13—C17	119.01 (18)	C31—C35—H35	116.6
C13—C14—C15	108.30 (19)	C35—C36—N6	121.4 (2)
C13—C14—H14	125.8	C35—C36—H36	119.3
C15—C14—H14	125.8	N6—C36—H36	119.3
C16—C15—C14	105.28 (19)	O2—N1—O3	123.55 (19)
C16—C15—H15	127.4	O2—N1—C6	120.06 (19)
C14—C15—H15	127.4	O3—N1—C6	116.38 (19)
C15—C16—O7	111.6 (2)	O5—N2—O6	122.62 (19)
C15—C16—H16	124.2	O5—N2—C12	117.32 (18)
O7—C16—H16	124.2	O6—N2—C12	120.07 (19)
C18—C17—C13	126.3 (2)	O9—N3—O8	122.83 (19)
C18—C17—H17	116.9	O9—N3—C18	119.96 (18)
C13—C17—H17	116.9	O8—N3—C18	117.21 (19)
C17—C18—N3	120.6 (2)	O11—N4—O12	122.61 (19)
C17—C18—H18	119.7	O11—N4—C24	120.2 (2)
N3—C18—H18	119.7	O12—N4—C24	117.14 (19)
C20—C19—O10	109.30 (18)	O15—N5—O14	123.11 (18)
C20—C19—C23	132.3 (2)	O15—N5—C30	117.66 (19)
O10—C19—C23	118.35 (19)	O14—N5—C30	119.2 (2)
C19—C20—C21	107.7 (2)	O17—N6—O18	122.96 (19)
C19—C20—H20	126.2	O17—N6—C36	119.78 (19)
C21—C20—H20	126.2	O18—N6—C36	117.3 (2)
C22—C21—C20	106.1 (2)	C1—O1—C4	105.67 (17)
C22—C21—H21	127.0	C10—O4—C7	105.69 (17)
C20—C21—H21	127.0	C16—O7—C13	105.73 (16)
C21—C22—O10	111.2 (2)	C22—O10—C19	105.77 (18)
C21—C22—H22	124.4	C25—O13—C28	106.26 (18)
O10—C22—H22	124.4	C31—O16—C34	105.61 (17)
			
O1—C1—C2—C3	0.3 (3)	C35—C31—C32—C33	−179.9 (2)
C1—C2—C3—C4	−0.4 (3)	C31—C32—C33—C34	0.1 (3)
C2—C3—C4—O1	0.4 (2)	C32—C33—C34—O16	−0.1 (3)
C2—C3—C4—C5	−179.7 (2)	C32—C31—C35—C36	178.6 (2)
C3—C4—C5—C6	−179.2 (2)	O16—C31—C35—C36	−1.2 (3)
O1—C4—C5—C6	0.7 (3)	C31—C35—C36—N6	179.89 (18)
C4—C5—C6—N1	179.79 (18)	C5—C6—N1—O2	−2.8 (3)
O4—C7—C8—C9	0.0 (2)	C5—C6—N1—O3	178.35 (19)
C11—C7—C8—C9	−179.6 (2)	C11—C12—N2—O5	178.7 (2)
C7—C8—C9—C10	−0.1 (3)	C11—C12—N2—O6	−1.9 (3)
C8—C9—C10—O4	0.1 (3)	C17—C18—N3—O9	2.5 (3)
C8—C7—C11—C12	177.2 (2)	C17—C18—N3—O8	−178.20 (19)
O4—C7—C11—C12	−2.4 (3)	C23—C24—N4—O11	−1.3 (3)
C7—C11—C12—N2	−179.95 (18)	C23—C24—N4—O12	178.77 (19)
O7—C13—C14—C15	−0.2 (2)	C29—C30—N5—O15	178.5 (2)
C17—C13—C14—C15	−179.2 (2)	C29—C30—N5—O14	−1.9 (3)
C13—C14—C15—C16	0.3 (2)	C35—C36—N6—O17	0.9 (3)
C14—C15—C16—O7	−0.3 (3)	C35—C36—N6—O18	−179.32 (19)
C14—C13—C17—C18	179.2 (2)	C2—C1—O1—C4	−0.1 (2)
O7—C13—C17—C18	0.3 (3)	C3—C4—O1—C1	−0.2 (2)
C13—C17—C18—N3	−178.25 (18)	C5—C4—O1—C1	179.84 (17)
O10—C19—C20—C21	0.4 (2)	C9—C10—O4—C7	0.0 (2)
C23—C19—C20—C21	−177.6 (2)	C8—C7—O4—C10	0.0 (2)
C19—C20—C21—C22	−0.5 (2)	C11—C7—O4—C10	179.69 (18)
C20—C21—C22—O10	0.3 (3)	C15—C16—O7—C13	0.2 (2)
C20—C19—C23—C24	174.8 (2)	C14—C13—O7—C16	0.0 (2)
O10—C19—C23—C24	−3.0 (3)	C17—C13—O7—C16	179.15 (18)
C19—C23—C24—N4	−179.47 (18)	C21—C22—O10—C19	−0.1 (2)
O13—C25—C26—C27	0.2 (2)	C20—C19—O10—C22	−0.2 (2)
C29—C25—C26—C27	179.0 (2)	C23—C19—O10—C22	178.11 (17)
C25—C26—C27—C28	−0.1 (3)	C26—C25—O13—C28	−0.3 (2)
C26—C27—C28—O13	−0.1 (3)	C29—C25—O13—C28	−179.25 (19)
C26—C25—C29—C30	−175.2 (2)	C27—C28—O13—C25	0.3 (3)
O13—C25—C29—C30	3.4 (3)	C32—C31—O16—C34	0.1 (2)
C25—C29—C30—N5	−179.79 (18)	C35—C31—O16—C34	179.92 (18)
O16—C31—C32—C33	−0.1 (2)	C33—C34—O16—C31	0.0 (3)

### Hydrogen-bond geometry (Å, °)

**Table:**

*D*—H···*A*	*D*—H	H···*A*	*D*···*A*	*D*—H···*A*
C1—H1···O10^i^	0.93	2.58	3.382 (3)	145
C16—H16···O13	0.93	2.60	3.370 (3)	141
C26—H26···O14^ii^	0.93	2.59	3.281 (3)	131
C28—H28···O8^iii^	0.93	2.59	3.405 (3)	146
C34—H34···O4	0.93	2.59	3.383 (3)	143

Symmetry codes: (i) *x*, *y*+1, *z*; (ii) −*x*+2, −*y*, −*z*+1; (iii) *x*−1, *y*, *z*.
